# Long-Term Follow-Up of Patients with West Nile Neuroinvasive Disease

**DOI:** 10.3390/v17070878

**Published:** 2025-06-23

**Authors:** Nataša Nikolić, Branko Milošević, Stojanović Miloš, Ljubisavljević Mila, Ivana Milošević, Nikola Mitrović, Jovan Malinić, Ana Filipović, Nevena Todorović, Uroš Karić, Boris Jegorović, Miloš Šabanović, Ivana Gmizić, Branko Beronja, Jasmina Poluga

**Affiliations:** 1 Clinic for Infectious and Tropical Diseases, University Clinical Center of Serbia, Bulevar Oslobođenja 16, 11000 Belgrade, Serbia; branko.milosevic@yahoo.com (B.M.); ivana.milosevic@med.bg.ac.rs (I.M.); nikola.mitrovic@med.bg.ac.rs (N.M.); jovan.malinic@gmail.com (J.M.); anafilipovic0211@gmail.com (A.F.); nevena.todorovic.1992@gmail.com (N.T.); uroskaric@gmail.com (U.K.); milossabanovic23@gmail.com (M.Š.); gmizic_ivana@yahoo.com (I.G.); 2 Faculty of Medicine, University of Belgrade, Dr Subotica 8, 11000 Belgrade, Serbia; milos.stojanovicc999@gmail.com (S.M.); mila.ljubisavljevic@gmail.com (L.M.); brankoberonj99@gmail.com (B.B.)

**Keywords:** West Nile virus, neuroinvasive disease, functional recovery, cognitive outcome

## Abstract

Human West Nile virus (WNV) infection is usually asymptomatic. Less than 1% of patients develop neuroinvasive disease (WNND) which may result in permanent neurological impairment. The aim of this study was to assess the functional and cognitive status of patients with WNND approximately one year after the onset of symptoms. This prospective observational cohort study involved patients with WNND. Patients’ functional and cognitive abilities one year post-infection were assessed by telephone interviews using the Modified Rankin Scale (mRS), Barthel Index, and Telephone Interview for Cognitive Status. Sixty-two participants were analyzed. All patients had encephalitis, and 7 (11.3%) also had acute flaccid paresis/paralysis (AFP). At discharge, 40 (64.5%) patients had no or minimal neurological deficit (mRS 0–1), and 14 (22.6%) were functionally dependent (mRS 3–5). One year later, 52 (83.9%) patients were functionally independent (mRS 0–2), none was severely dependent (Barthel index 0–60), and 50 (90.9%) had a Barthel index score of 91–100. Among 14 functionally dependent patients at discharge, 3 (21.4%) remained functionally dependent one year later. During the follow-up, 7 (11.3%) patients died. No significant difference was observed in the fatality rate between patients with and without AFP, mRS 3–5 at discharge, or age over 65. The most common persistent symptoms were muscle weakness, walking instability, and issues with focus and memory. Using TICS, it was found that 33/55 patients (60%) had unimpaired and 2 (3.6%) had moderately or severely impaired cognitive status. The long-term prognosis after WNV encephalitis is satisfying. The majority of patients reached functional independence and 60% had unimpaired cognitive status.

## 1. Introduction

West Nile virus (WNV) is a single-stranded RNA arbovirus which belongs to the Flaviviridae family. It was discovered in Uganda in 1937 and was first detected in Europe in the second half of the 20th century [1,2,3,4]. In the following period, it became the most common cause of epidemic viral encephalitis in Europe [5]. The first human cases were documented in Serbia in 2012, and additional cases are reported every year [5,6]. The majority of human cases of WNV infection are asymptomatic, and less than 1% of infected individuals acquire the neuroinvasive form of the illness, known as West Nile neuroinvasive disease (WNND) [2,3,4]. Meningitis, encephalitis, and/or acute flaccid paresis/paralysis (AFP) are possible presentations of WNND. WNE and/or AFP may result in permanent neurological impairment [2,3,7]. Numerous reports exist on the acute phase of WNND, but few about the long-term follow-up of patients with this form of the illness. The aim of this study was to assess functional status and persistent cognitive deficit in patients with WNND approximately one year after acute disease.

## 2. Materials and Methods

### 2.1. Study Design

This is a prospective observational cohort study involving patients diagnosed with WNND who were hospitalized at the University Clinical Center of Serbia’s Clinic for Infectious and Tropical Diseases between 1 June 2022, and 31 October 2022. A year (between 11 and 15 months) after the acute illness, the patients’ functional and cognitive abilities were assessed using telephone interviews that were conducted between 1 August 2023, and 30 November 2023.

The study excluded patients who refused to participate, had terminal malignancies, or had other conditions that could affect their functional or cognitive status. It also excluded patients we were unable to reach using the available contact information. Moreover, West Nile meningitis (WNM) is usually a mild disease with good prognosis that results in full recovery with no permanent neurological deficits; thus, individuals who presented with WNM were also omitted from the analysis [8].

### 2.2. Diagnostic Criteria and Case Definition

Diagnostic criteria for probable and confirmed cases of West Nile virus infection were established according to the European Union case definition [9]. These included at least one of the following clinical criteria: the presence of fever, encephalitis, and/or meningitis, as well as laboratory and epidemiological criteria. At least one of the following laboratory criteria was required for case confirmation: isolation of WNV from blood or cerebrospinal fluid (CSF), detection of WNV nucleic acid in blood or CSF, WNV-specific antibody response (immunoglobulin M; IgM) in CSF, and/or WNV IgM high titer, detection of WNV IgG, and confirmation by neutralization. The presence of only WNV-specific antibody response in serum was considered laboratory criteria for probable case. Epidemiological data that were considered as criteria for WNV infection were potential animal to human transmission (residing, having visited or having been exposed to mosquito bites in an area where WNV is endemic in horses or birds) and/or human to human transmission (vertical transmission, blood transfusion, transplants). A probable case of human WNV infection was established in the presence of clinical criteria, laboratory test for probable case, and at least one epidemiological criterion. Any person meeting laboratory criteria for case confirmation was considered a confirmed case of WNV infection.

In order to assess for the presence of IgM and IgG WNV-specific antibodies, acute phase cerebrospinal fluid (CSF) and acute and convalescence phase serum samples were obtained on the first day of hospitalization and within 10–14 days of the onset of symptoms. Before testing, the samples were kept between −25 and 4 °C during transportation. The National Reference Laboratory for Arboviruses of the Institute of Virology, Vaccines and Sera “Torlak” in Belgrade performed the enzyme-linked immunosorbent assay (ELISA) analysis on the samples (Anti-West Nile Virus ELISA (IgM) and Anti-West Nile Virus ELISA (IgG), EUROIMMUN, Medizinische Labordiagnostika AG, Lübeck, Germany). Every patient who was enrolled had sterile CSF cultures, negative CSF PCR analyses for enteroviruses, human immunodeficiency virus, cytomegalovirus, Epstein–Barr virus, and herpes viruses, as well as negative sera and CSF tests for tick-borne encephalitis and Lyme disease serology. None of the patients had a history of flavivirus vaccination.

WNND was diagnosed in patients who presented with encephalitis, meningitis, or acute flaccid paralysis (AFP). Meningitis was defined as the presence of fever, and/or headache, nuchal rigidity, Kernig’s sign or Brudzinski signs, photophobia or phonophobia, and the presence of CSF pleocytosis (>5 leucocytes/mm^3^; norm: 0–5 leucocytes/mm^3^), elevated protein levels (>0.45 g/L; norm: 0.15–0.45 g/L), and normal (2.6–3.1 mmol/L, 50–60% of serum glucose levels) or mildly decreased (2.4–2.6 mmol/L) CSF glucose level [10,11].

Fever, encephalopathy (lowered or altered level of consciousness, lethargy, or personality change), focal neurological signs (weakness, cranial nerve palsies), seizures, movement disorders (tremor, parkinsonism, ataxia), and CSF findings similar to those in patients with WNV meningitis were all considered indicators of WNV encephalitis. [10,11].

AFP was defined as the sudden onset of paralysis or limb weakness that progressed over a 48 h period. Limb weakness in WNV AFP is characterized by at least two of the following characteristics: asymmetry, areflexia/hyporeflexia of affected limbs, absence of pain and sensitive deficit, electrodiagnostic studies consistent with an anterior horn cell lesion, CSF pleocytosis (>5 leucocytes/mm^3^), and elevated protein levels (>0.45 g/L) [10].

Patients who met the WNND criteria and had cerebellar ataxia, wide-based gait, dysmetria, and/or cerebellar speech disorders were diagnosed with cerebellitis, whereas patients with cerebellar lesion and/or signs of brainstem involvement were diagnosed with rhombencephalitis.

### 2.3. Data Collection and Instruments Used for Patients’ Evaluation

Patients’ demographic characteristics, comorbidity, clinical presentation, and functional outcome at hospital discharge using modified Rankin scale (mRS) [12] were recorded using medical records.

The condition of patients approximately one year after acute disease was assessed by telephone interview, after obtaining the verbal informed consent of each patient. Telephone interview was conducted by experienced clinicians who treated the patients in the acute phase of the disease.

The Modified Rankin Scale, the Barthel Index, and the Telephone Interview for Cognitive Status (TICS) were used to evaluate the functional and cognitive statuses of patients one year after their hospital stay.

The Modified Rankin Scale (mRS) is a clinician-reported measure of disability, commonly used to assess the degree of neurological impairment [12]. Patients with mRS 0 and 1 had no or minimal neurological deficit, respectively. Those who scored 0–2 were classified as functionally independent, 3–5 as functionally dependent, and a score of 6 by mRS represented fatal outcome [12].

The Barthel index is a simple index of independence useful in scoring the ability of patients with neurological and neuromuscular disorders to care for themselves as well as in following their improvement during rehabilitation. According to the Barthel index, patients with a score of 0–20 were classified as totally dependent, 21–60 as severely dependent, 61–90 as moderately dependent, 91–99 as slightly dependent, and 100 as independent [13].

The TICS total score provides a measure of global cognitive functioning and can be used to monitor changes in cognitive functioning over time [14]. The impairment ranges have been shown to adequately distinguish between normal participants and patients with cognitive impairment. It consists of questions regarding basic cognitive functions such as memory, focus, concentration, speech, etc. Scores range from 0 to 41. Patients scoring 33–41 are considered to have unimpaired cognitive status, those scoring 26–32 ambiguous, a TICS score 21–25 suggests mild cognitive impairment, and less than 22 indicates moderate to severe impairment [14].

### 2.4. Statistical Analysis

Descriptive and analytical statistics were used to analyze all of the collected data. The association of several variables with functional outcome and cognitive status one year after discharge was analyzed using univariate analysis. Chi-square test and Fisher’s exact test were applied for categorical variables and Student’s *t*-test for continuous variables. The Statistical Package for the Social Sciences (SPSS) software for Windows (version 17.0) was used for statistical analysis. Statistical significance was set at 0.05.

### 2.5. Ethical Approval and Consents

This study was approved by the Ethical Board of University Clinical Center of Serbia’s Clinic for Infectious and Tropical Diseases (number 23/07). No written consent forms were available because the study was designed to collect data through telephone interviews; nevertheless, before responding to certain questions, all patients or their caregivers provided their informed verbal consent to participate.

## 3. Results

### 3.1. Patients’ Characteristics

Between 1 June 2022 and 31 October 2022, a total of 135 patients with WNND were hospitalized at the University Clinical Center of Serbia’s Clinic for Infectious and Tropical Diseases. Among them, 106 (78.5%) patients presented with encephalitis. Of these, 29 (27.4%) died during the acute phase, while 77 (72.6%) were discharged. Of the 77 patients with encephalitis who survived, 62 were enrolled in the follow-up study. One patient (1.3%) was excluded due to a concurrent terminal cancer, and 14 patients (18.2%) could not be contacted using the available information.

The majority of the patients, 42 (67.7%), were over 65 years old. The most common comorbidity was hypertension, which was present in 40 (64.5%) patients (Table 1).

All of the analyzed patients had encephalitis. Of them, 49 (79.0%) presented as rhombencephalitis and among them, 45 had clinical signs of cerebellitis. Seven (11.3%) patients had AFP. Monoparesis was present in 4 (6.5%) and paraparesis in 3 (4.8%) patients.

Among these 62 patients, at discharge, 40 (64.5%) patients had no or minimal residual neurological deficit using mRS (mRS 0–1), 8 (12.9%) patients had mRS 2 and were also functionally independent, and 14 (22.6%) patients were functionally dependent (mRS 3–5).

### 3.2. Functional Outcome at One-Year Follow-Up

During the one-year period after discharge, 7 (11.3%) patients died (mRS 6). Five of them died during the first month upon discharge. COVID-19 was the cause of death in two of them, and Clostridium difficile infection and pulmonary embolism in one patient each. Among these five patients, we were unable to determine the probable reason for one patient’s death. Comorbidities caused the death of the two remaining patients, 9 and 11 months following acute WNND.

No significant difference was observed in the fatality rate between patients with and without AFP, mRS 3–5 at discharge, or age over 65 (*p* = 1.000; *p* = 0.153; and *p* = 0.412, respectively).

The functional status at discharge of those 55 patients who were alive at one-year follow-up using mRS is shown in Table 2. Among them, 48 (87.3%) patients had mRS 0–1 one year following hospitalization.

Barthel index scores were calculated only at the time of follow-up assessment. Fifty (90.9%) patients had Barthel index 91–100, suggesting minimal disability or no remaining neurological deficit (Table 2).

Eleven patients had data obtained regarding the dynamics and progression of their functional recovery. Of these, three, three, and five patients said it took one, three, and six to twelve months, respectively, to recover to the final level. Among 14 functionally dependent patients at discharge, 3 (21.4%) remained functionally dependent one year later, and the same number of them died.

### 3.3. Self-Reported Symptoms at One-Year Follow-Up

One year after the infection, the most common self-reported symptoms were depression, muscle weakness, walking instability, and issues with focus and memory. Three patients experienced de novo convulsions during WNND, and one of them continued to have them a year later. Six patients, three of whom presented as AFP, reported having persistent muscle weakness. The remaining 4 patients with AFP at presentation regained full premorbid muscle strength. In 9 out of 23 patients (39.1%) who had walking instability at discharge, the condition persisted one year later (Table 3).

### 3.4. Cognitive Outcome at One Year Follow-Up

Using TICS to measure cognitive status, 33 out of 55 patients (60%) had unimpaired cognitive status, whereas 2 (3.6%) had moderately or severely impaired cognitive status. One patient (1.8%) had TICS in the range 21–25, while 19 (34.5%) had TICS 26–32. There was no statistical difference in cognitive status between patients regarding mRS at discharge, mRS one year later, the presence of AFP, rhombencephalitis, or age over 65 (Fisher’s exact test; *p* = 0.376; *p* = 0.851; *p* = 0.874; *p* = 0.206; and *p* = 0.383, respectively) (Table 4).

Additionally, there was no significant difference between patients with and without AFP in terms of the functional outcome measured one year after infection using the mRS. On the other hand, significant difference in functional outcome one year post-infection assessed with the Barthel index was found between those who were functionally dependent and independent at discharge (Fisher’s exact test; *p* = 0.03) (Table 5).

In order to exclude potential influence of other confounding variables, multivariate analysis was performed. After adjustment for age over 65, presence of hypertension, previous stroke, diabetes, the presentation of AFP and/or rhombencephalitis, mRS 3–5 was found to be the independent predictor for Barthel index at one-year follow-up (*p* = 0.005).

Among the patients studied, 14 had been employed when the illness occurred. Following the disease, they all continued to work at their prior positions.

## 4. Discussion

Human WNV infection is usually asymptomatic [2,3,4,5]. Older age is one of the most significant risk factors for neuroinvasive disease which develops in less than 1% of those infected [2,3,7]. In the present study, 67.7% of patients were over 65 years old. Other risk factors include immunosuppression and comorbidities affecting the integrity of blood–brain barrier, among which hypertension and diabetes were the most common in our study population [2,3,7,15].

In the present study, the case fatality rate during acute WNE was 27.5%. This is in accordance with the results of Anastasiadou et al. who reported the in-hospital fatality rate of 25.8% in patients with WNND [16]. Other authors reported fatal outcome in 10–30% of patients with WNE [17,18,19,20].

During the one-year follow-up, 7 (11.3%) patients with previous WNE died. For five of them, death occurred during the first month after hospital discharge, suggesting that death was either a direct consequence of WNE or the result of complications occurring during hospitalization. Prolonged hospital stay and ICU treatment increase the risk of healthcare-associated infections which are significant predictors of fatal outcome both during and after hospitalization.

According to mRS, 87.3% of the individuals who survived the one-year follow-up period showed minimal or no persisting neurological deficit at check-up. When functional status was assessed using the Barthel index, the percentage of almost totally functionally recovered patients even slightly increased to 90.9%, suggesting its potentially lower sensitivity to minor motor deficits. These findings are consistent with those of Hawkes et al., who found that 80% of patients were functionally independent (mRS 0–2) at least one year after acute disease [21]. In contrast, a study of WNV-infected patients in New York found that only 37% fully recovered one year after acute illness, but this “total” recovery comprised not only functional ability but also cognitive and physical aspects. When taking into consideration only functional outcome, 57.1% achieved functional independence. Difficulty with walking was present in 49% and 44% suffered from muscle weakness [19]. In the study by Sejvar et al. five out of 16 patients with WNE had totally recovered at the 8-month follow-up visit, and one patient still required invasive ventilatory support [22]. Among 22 patients with WNND from the Greece cohort, 31.8% recovered completely, with a mean recovery duration of 6.26 months [16]. All of the abovementioned studies included fewer patients compared to the present study, and the difference in the number of involved subjects might explain the divergence in the outcomes. Furthermore, a notable percentage of our patients with WNE had rhombencephalitis/cerebellitis, which was already demonstrated to be a frequent presentation in Serbian patients with good outcome at discharge [6]. The high percentage of these patients who had satisfactory functional outcome at one year follow-up (41/43 had mRS 0–2) could explain the better overall outcomes in our study population. In addition, functional outcome in different studies was evaluated using different scales and instruments which might have different sensitivity for minor motor deficits.

Another potential reason for better functional recovery and the significant proportion of patients with rhombencephalitis/cerebellitis in the present study could be the lineage of the virus. Specifically, so far, lineage 2 was isolated in Serbian patients [23]. Phylodynamic analyses of the Balkan clade indicated the potential for an increase in genetic diversity and structure of virus populations [23]. Although lineage 2 was also isolated throughout Europe, Asia, and America, differences among strains might be linked to specific clinical presentations and outcomes.

Although the findings of different authors vary, it appears that patients with WNND and WNE have a better long-term functional outcome than those with Herpes simplex encephalitis (HSE), who achieve complete recovery in 14–50% after at least one year [24,25,26,27]. Nevertheless, this impression should be taken with caution, due to the lack of direct comparison in a single cohort study and different study designs. In the study of Jouan et al., 87 % of the patients with HSE were alive after one year but half of them had moderate to severe disability [24]. A review article on outcome and sequalae of infectious encephalitis which was published in 2024 reported that 30–70% of HSE survivors have persisting symptoms and sequelae with significant consequence [28]. Cognitive decline in several domains has been reported in most patients and has a major impact on functional outcomes, as well as behavioral changes which might be observed in 40–50% of survivors [28]. On the other hand, persisting motor or coordination deficits are less common than cognitive and behavioral symptoms after HSE [28].

In addition to objective assessments, self-reported symptoms were used to assess persistent neurological deficits. Ataxia and gait instability were the most prevalent, and these disorders were also the most frequently observed abnormalities during acute illness [29]. Often, the patient’s functional independence was unaffected by this ongoing gait instability, which led to favorable mRS and Barthel index scores. Ataxia usually resolves after 1–3 weeks, spontaneously or after corticosteroid therapy, but might persist for more than a year [30]. According to the literature data, 15–60% of patients had balance issues at least a year after acute disease [20,22,31]. The etiology of this deficit is probably multifactorial, including deficits in motor, neurosensory, and vestibular function [31].

Another frequently reported persistent symptom among people who survive WNE is tremor. While tremor may disappear while a patient is still in the hospital, there have also been documented cases of it lasting up to a year [21,32]. In the previously mentioned Houston study, tremor persisted in 5%, 2%, and 1% of patients at 1-year, 2-year, and 5-year follow-up, respectively [20]. Carson et al., and Anastasiadou et al. reported tremor in 20% and 18.2% of patients during follow-up, respectively. In the present study, tremor persisted in 3.8% of patients [16,32].

Patients with WNND have been known to experience movement problems both during the acute illness and in the follow-up period. Based on MRI and postmortem data, it is believed that these symptoms are caused by inflammation in the basal ganglia and cerebellum [18].

During and following acute WNE cranial nerve palsies may also be observed in up to 70% of patients [33]. Most of them have facial nerve involvement. After a year, the patient who had bilateral peripheral facial nerve palsy and had been assessed in this study had fully recovered.

Recovery of AFP is usually prolonged and often incomplete. In the New York cohort, 12 months following an acute WNV infection, 15 out of 34 patients had muscle weakness; in the Houston trial, 103 participants experienced paralysis at the 1- and 2-year check-ups, respectively [19,20]. Strength recovery mostly occurs during first 6–8 months following weakness onset, with a subsequent plateauing during which additional strength recovery may happen, but to a less notable degree [34,35]. In the present study, one year post-infection, muscle weakness was still observed in 3 of 7 patients who presented with AFP. The other 4 patients recovered complete strength, among which 1 patient had severe paraparesis and was functionally dependent at discharge. This observation suggests that the degree of muscle strength recovery is not associated with the severity of initial weakness, and according to the literature, even patients with severe quadriparesis may recover completely.

Three additional patients who did not have an established diagnosis of AFP during their acute illness reported having muscle weakness at the one-year check-up. However, since this was a self-reported symptom and not the result of an objective neurological examination, it is possible that the patient did not actually have limb paresis but rather had a subjective impression of it.

Out of the patients analyzed, one (1.8%) had de novo convulsions a year after the onset of acute illness. Murray et al. similarly observed a low frequency of post-WNE convulsions, occurring in only 1% at periods of 1-, 2-, and 5-year follow-up [20]. On the contrary, convulsions were seen considerably more often (in 28.1%) in the study by Shmidt et al., which evaluated the long-term prognosis and sequela in patients with encephalitis of undetermined etiology [36]. Seizures were found in 14% of 34 patients in a case series addressing sequalae of HSE [37]. More frequent seizures in patients after HSE might be explained by the fact that HSE affects mainly temporal and orbitofrontal regions which are epileptogenic. On the contrary, WNV affects the basal ganglia, spinal cord, and cerebellum, resulting in the abovementioned sequalae. The HSV tropism towards temporal lobe, including hippocampus and amygdala, and cortical necrotizing lesions in HSE also explain more frequent presence of cognitive deficit, memory loss, speech disorders, and behavioral changes following HSE in comparison to WNE.

Loss of taste, tinnitus, and partial hearing loss were observed in 1 (1.8%) patient each. Similarly, three patients (13.6%) in the Greek cohort reported hearing loss at a 16-month follow-up [16]. In contrast, Weatherhead et al.’s study found that 46% of patients had hearing abnormalities [32]. However, these results should be interpreted cautiously because a significant portion of the patients were elderly and may have age-related hearing loss [32]. Additionally, some of the patients had prior hearing loss.

The ability of the virus to remain in several tissues, including the central nervous system, as evidenced by an experimental study involving mice, might be responsible for the persistence of neurological deficit after WNE, along with the damage induced by inflammation during acute illness [38].

Encephalitis of various etiologies might result not only in functional dependency, but also in cognitive and emotional dysfunction such as memory loss, concentration problems, depression, etc. These mental changes might affect quality of life and social and emotional functioning. Nine months following acute WNE, 42% of patients in research by Haaland et al. had memory and concentration problems, and 65% of patients had abnormal TICS [30]. In the present study, memory and focus problems were recorded less often, in 14.5% and 7.3% of patients, respectively. Abnormal TICS was present in 39.9% of patients, but poor TICS 0–20 was observed in only 3.6%.

Similar findings were presented by Bogovič et al., who examined cognitive dysfunction one year following tick-borne encephalitis and discovered that 16.9% of patients had memory problems [39]. In comparison, Mailles et al. reported long-term memory disturbances in 30.3% of patients with the history of herpes simplex encephalitis [27].

Another study assessing cognitive impairment among 49 patients recovering from WNND and WNF showed that cognitive ability is mostly within normal limits, and abnormalities were found in the domain of manual dexterity and motor speed [31]. Similar findings were observed in a WNV-infected cohort from Colorado [40]. The majority of research on cognitive impairment following WNV infection indicates that while subjective symptoms are rather common, they do not always correlate with poor performance on objective tests. The same is observed for functional status. It is possible that the persistent symptoms are sometimes subtle and do not affect functionality, but it should also be considered whether patients sometimes overestimate their subjective symptoms and condition.

All these data suggest that patients surviving WNE have more favorable long-term functional, as well as cognitive status and thus, probably better quality of life, compared to patients who suffered encephalitis of another etiology.

Upon analyzing risk factors for ongoing functional dependency one year post-WNE, our study revealed that none of them was a significant predictor. Literature data demonstrated that older age was the major risk factor for poor long-term functional outcome and persistent symptoms [19,20,32]. The fact that the majority of our patients were over 65 years old might explain this result.

This is the first study in Serbia addressing long-term follow-up of patients with WNE regarding functional and cognitive status. It provides valuable information about the potential prognosis of these patients as well as issues they may encounter after being discharged. It should come as no surprise that this study is one of the biggest regarding the subject, given that Serbia is one of the European countries most impacted by WNV infection in recent years. Furthermore, the data were obtained using both objective scales and self-reported symptoms, thus providing more realistic and objective results.

This study also has potential limitations. The fact that the information about persistent signs and symptoms was gained by telephone interview and not by an objective neurological examination might affect the results. Furthermore, we were unable to reach all of the patients, and the outcomes of those who were not evaluated could have an impact on the overall long-term prognosis. Finally, the check-up of patients at 1, 3, and 6 months after acute illness would provide more information about the course and dynamics of patient recovery.

## 5. Conclusions

According to all the abovementioned points, WNE might result in long-term motor and cognitive disabilities which can affect the quality of life of patients and their families. The overall recovery and long-term prognosis after WNE are satisfying; the majority of patients reached functional independence and, compared to the literature data, prolonged cognitive deficit was observed less frequently than in patients who had HSV encephalitis, but this should be considered cautiously. The most common persistent symptoms were gait instability and tremor. Given the potential for both permanent and long-term impairment following WNND and the lack of causal therapy, it is imperative to underscore the significance of preventative measures and effective mosquito control.

## Figures and Tables

**Table 1:** Demographic characteristics and comorbidities in patients with WNE (n = 62); SD—standard deviation.

Characteristic	N (%)
Male	42 (67.7)
Age (years ± SD)	67.1 ± 13.2
Age > 65 years	42 (67.7)
Rural area	24 (38.7)
Comorbidities	
Hypertension	39 (62.9)
Diabetes	16 (25.8)
Coronary disease	9 (14.5)
Previous stroke	6 (9.7)
Malignancy	4 (6.5)

**Table 2:** Functional outcome of survived patients with WNE with/without AFP at discharge and one year after acute disease using mRS and the Barthel index (n = 55).

Functional Outcome	At Hospital Discharge	At One Year Follow-Up
mRS		
0–1	38 (69.1%)	48 (87.3%)
2	7 (12.7%)	4 (7.3%)
3–5	10 (18.2%)	3 (5.5%)
Barthel index		
0–20	NA	0
21–60	NA	0
61–90	NA	5 (9.1%)
91–99	NA	36 (65.5%)
100	NA	14 (25.4%)

mRS—modified Rankin Scale; NA—not available.

**Table 3:** Persistent self-reported symptoms in patients one year after WNE (n = 55).

Symptom	N (%)
Difficulties with focus and concentration	4 (7.3)
Memory difficulties	8 (14.5)
Feeling depressed	4 (7.3)
Tremor	2 (3.6)
Convulsions	1 (1.8)
Vertigo	3 (5.5)
Gait instability	9 (16.4)
Muscle weakness	6 (10.9)
Headache	3 (5.5)
Tinitus	1 (1.8)
Disgeusia	1 (1.8)
Behavior difficulties	0 (0)

**Table 4:** Distribution of patients’ characteristics according to TICS categories(n = 55).

Risk Factor	TICS 0–20 N = 2	TICS 21–25 N = 1	TICS 26–32 N = 19	TICS 33–41 N = 33	*p*
Age > 65	2 (100%)	1 (100%)	14 (73.7%)	19 (57.6%)	0.383
AFP	0 (0%)	0 (0%)	2 (10.5%)	5 (15.2%)	0.874
Rhombencephalitis/cerebellitis	2 (100%)	0 (0%)	16 (84.2%)	25 (75.8%)	0.206
mRS 3–5 at discharge	1 (50.0%)	0 (0%)	5 (26.3%)	4 (12.1%)	0.376
mRS 3–5 one year post-infection	0 (0%)	0 (0%)	2 (10.5%)	2 (6.1%)	0.851

**Table 5:** Univariate analysis of different characteristics of patients according to their functional status one year after WNE and/or AFP using mRS and Barthel index (BI) (n = 55).

Risk Factor	mRS 0–2 N = 52	mRS 3–5 N = 3	*p*	BI 61–90 N = 5	BI 91–100 N = 50	*p*
Age > 65	33 (63.5%)	3 (100.0%)	0.544	3 (60.0%)	33 (66.6%)	0.570
AFP	6 (11.5%)	1 (33.3%)	0.341	1 (20.0%)	6 (12.0%)	0.508
Rhombencephalitis/cerebellitis	41 (80.4%)	2 (66.6%)	0.530	3 (60.0%)	40 (80.0%)	0.298
mRS 3–5 at discharge	8 (15.4%)	2 (66.6%)	0.082	4 (80.0%)	6 (12.0%)	0.030

## Data Availability

Data is unavailable due to privacy or ethical restrictions.
